# Omicron Sub-Lineage BA.5 and Recombinant XBB Evasion from Antibody Neutralisation in BNT162b2 Vaccine Recipients

**DOI:** 10.3390/microorganisms11010191

**Published:** 2023-01-12

**Authors:** Martina Brandolini, Giulia Gatti, Laura Grumiro, Silvia Zannoli, Valentina Arfilli, Monica Cricca, Giorgio Dirani, Agnese Denicolò, Maria Michela Marino, Martina Manera, Andrea Mancini, Francesca Taddei, Simona Semprini, Vittorio Sambri

**Affiliations:** 1 Unit of Microbiology, The Greater Romagna Area Hub Laboratory, 47522 Cesena, Italy; 2 Department of Experimental, Diagnostic and Specialty Medicine (DIMES)—Alma Mater Studiorum, University of Bologna, 40138 Bologna, Italy

**Keywords:** SARS-CoV-2, XBB recombinant, neutralising antibody response, mRNA-vaccine, immune escape

## Abstract

The recent emergence of a number of new SARS-CoV-2 variants resulting from recombination between two distinct parental lineages or sub-lineages within the same lineage has sparked the debate regarding potential enhanced viral infectivity and immune escape. Among these, XBB, recombinant of BA.2.10 and BA.2.75, has caused major concern in some countries due to its rapid increase in prevalence. In this study, we tested XBB escape capacity from mRNA-vaccine-induced (BNT162b2) neutralising antibodies compared to B.1 ancestral lineage and another co-circulating variant (B.1.1.529 BA.5) by analysing sera collected 30 days after the second dose in 92 healthcare workers. Our data highlighted an enhanced and statistically significant immune escape ability of the XBB recombinant. Although these are preliminary results, this study highlights the importance of immune escape monitoring of new and forthcoming variants and of the reformulation of existing vaccines.

## 1. Introduction

The implementation of global vaccination campaigns has proved to be an effective countermeasure to stem the spread of SARS-CoV-2, reducing the number of severe cases, hospitalisation rates and attributable mortality. Among the numerous vaccines which have been developed, Pfizer-BioNTech BNT162b2 received emergency authorisation in late 2020, and has since been widely used. Alongside this, other mRNA-based, viral vector-based, inactivated and recombinant subunit vaccines have also been developed. Among these, mRNA-vaccines are based on the ancestral Spike protein gene sequence [1,2,3]. 

In the last two years, the pandemic has continued and SARS-CoV-2 epidemiology has been characterised by a continuous evolution, with the periodic emergence of extremely diverse viral variants (also named Variants of Concern, or VOCs for short). These have raised the concern of the scientific community not only for their relatively higher transmissibility and virulence, and hence associated disease severity, but also for their ability to reduce the efficacy of therapeutics and escape infection- or vaccination-induced antibody response [4,5,6]. The emergence of VOCs has been linked to increased seroprevalence, which has been hypothesised to be a key factor behind SARS-CoV-2 evolution, capable of exerting a selective pressure toward an augmented viral fitness over preceding variants in the context of an immune population. This hypothesis was later experimentally demonstrated in vitro [7,8]. 

These evolutionary characteristics have prompted intensively debated questions and speculations, primarily regarding how vaccines will contribute to the emergence of new variants. Moreover, as many vaccines are based on the ancestral Spike protein gene sequence, they elicit a relatively “narrow-spectrum” immune response, which can be easily and rapidly eroded by viral evolution. In fact, there is emerging evidence that the high mutation rate of the S gene constitutes a breeding ground for immune escape mechanisms, reducing the neutralising potential of antibodies produced in vaccinated subjects. In this context, what is the ability of a specific vaccine to respond to forthcoming variants? Many studies have sought an answer, highlighting how mutations (especially on the Spike) can disrupt key antibody epitopes, thus conferring immune evasion properties to VOCs [9]. 

More recently, the changing epidemiology alongside prolonged high-incidence waves and simultaneous attacks of different variants have demonstrated the ability of SARS-CoV-2 to undergo recombination events, which can occur between different lineages or sub-lineages [10,11]. These recombinant variants, at least theoretically, may have slightly different biological characteristics compared to the parental strains, including a different transmission rate, more severe outcomes and/or a different susceptibility to antibody neutralisation [12]. While these assumptions have been proposed based on bioinformatics’ analysis, they still need, for the most part, in vitro and in vivo validation. Presently, the XBB recombinant variant, a hybrid of BA.2.10 and BA.2.75, which was first detected in August 2022, has given rise to apprehension due to its theoretically augmented infectivity and immune escape ability [13]. In this study, we tested the ability of this recombinant to evade vaccine-induced antibody neutralisation compared to the ancestral lineage (B.1) and another co-circulating variant (B.1.1.529 BA.5).

## 2. Materials and Methods

### 2.1. Cell Culture Conditions

Vero E6 cell cultures were maintained in Minimum Essential Medium (MEM) with 10% heat inactivated foetal bovine serum (FBS), 100 U/mL penicillin, 100 μg/mL streptomycin and 2 mM L-glutamine added, as recommended. Cells were incubated at 37 °C in a 5% CO_2_ atmosphere-enriched and humidified chamber until use [14].

### 2.2. Virus Sequencing, Propagation and Titration

Viral strains were isolated on cell culture from residual clinical specimens (nasopharyngeal swabs) provided to the Unit of Microbiology, Greater Romagna Area Hub Laboratory, Cesena, Italy, for routine diagnostic purposes and randomly selected for sequencing as part of a national project to monitor temporal trends of distribution and prevalence of SARS-CoV-2 viral variants in Italy, as previously described [8]. Each isolated strain was thereafter sequenced to reconfirm the lineage identification provided for the clinical specimen. Library preparation was performed using the CleanPlex SARS-CoV-2 Flex Research and Surveillance NGS Panel (Paragon Genomics, Inc., Hayward, CA, USA). Paired-end and dual-indexed sequencing was carried out on a MiSeq instrument (Illumina, San Diego, CA, USA) [15]. Once isolated, viral strains were then titrated using the endpoint dilution method and viral titres were calculated with the Reed and Muench formula based on eight replicated for dilution and expressed as TCID_50_/mL [16,17].

### 2.3. Convalescent Sera and Neutralisation Test

All sera samples were residual clinical specimens collected from healthcare workers in order to monitor their humoral immune response after vaccination with Comirnaty BNT162b2, BionTech/Pfizer. Sera collected one month after the second dose of vaccine were included in the study. None of the enrolled subjects reported a previous SARS-CoV-2 infection. The samples were provided to the Unit of Microbiology, Greater Romagna Area Hub Laboratory, Cesena, Italy, to measure specific anti-S1/S2 IgG levels and determine neutralising efficacy against wild-type lineage B.1. Before being included in this study, all samples underwent an anonymization procedure, in order to adhere to the regulations issued by the local Ethical Board (AVR-PPC P09, rev.2; based on Burnett et al., 2007 [18]).

IgG for S1/S2 subunits of SARS-CoV-2 spike were measured with a commercial chemiluminescence-based immunoassay (CLIA), LIAISON SARS-CoV-2 S1/S2 IgG (DiaSorin, Vicenza, Italy) [19]. Positive sera were selected for a neutralisation test on cell culture. Sera samples were tested at a starting dilution of 1:10 and then further diluted 1:2 until 1:5120. Microneutralisation assay on cell culture was performed as previously described [8]. Each serum dilution was incubated on Vero E6 cell cultures with 100 TCID50/mL of the considered viral lineages. Neutralisation titres were determined after 72h of incubation. Every serum sample was tested in duplicate. Absence or presence of cytopathic effect at each dilution was assessed by comparison of each well with virus control and cell control wells. The neutralisation titre (NT) was defined as the reciprocal of the highest serum dilution capable of inhibiting the appearance of a visible cytopathic effect [20]. Sera samples were grouped based on their neutralising titre against B.1 lineage. In total, for titres ranging from 20 to 1280, we selected 12 sera for each class, while only 8 sera with a titre of 2560 were available and therefore included. Sera with titres lower than 20 (NT ≤ 10) were not considered. These sera were retested with B.1.1.529 BA.5 lineage and XBB recombinant in order to quantify the enhanced resistance to antibody neutralisation of these mutated variants.

All activities involving the manipulation of infectious virus were performed in a Biological Safety Level 3 (BSL-3) facility at the Unit of Microbiology, Greater Romagna Area Hub Laboratory, Cesena, Italy, in compliance with appropriate containment rules.

### 2.4. Statistical Analysis

Statistical analysis was carried out using OriginPro 8.5 (OriginLab Corporation, Northampton, MA, USA). For statistical comparison of unpaired sets of data, a non-parametric Mann–Whitney test was used, based on the non-normal distribution of the data. Neutralisation titres against the three viral variants considered were compared using a one-way repeated-measures ANOVA test. The correlation between neutralising titres against the three variants and anti-S1/S2 IgG values for each volunteer were analysed using a non-parametric two-tailed Spearman’s rank correlation. For every statistical test, two levels of statistical significance were considered (*p* < 0.05 **, *p* < 0.01 ***) to measure the significance of the obtained result.

## 3. Results

Using a commercial CLIA-based assay and a live-virus-based microneutralisation assay, we determined the anti-S1/S2 IgG value and neutralisation titre in 92 Italian healthcare workers (66 females and 26 males), with a mean age of 43 years (standard deviation = 10.9 years, median age = 42 years, IQR (interquartile range) = 34.5–52), who had been vaccinated with two doses of BNT162b2 (Pfizer–BioNTech) and had received the second dose 30 days before blood sampling. The results of the Spearman correlation indicated that there is a very small but non-significant negative relationship between age and anti-S1/S2 IgG, as well as between age and neutralising titres against the considered lineages; the *p* value never reached the lower threshold of statistical significance (*p* > 0.05). On the contrary, a non-parametric Mann–Whitney test for unpaired samples showed a significant difference between male and female participants in anti-S1/S2 IgG values (*p* < 0.05, specifically *p =* 0.021) and neutralising titre against lineage BA.5 (*p <* 0.05, specifically *p* = 0.024), but not in neutralising titre against lineage B.1 (*p* > 0.05, *p =* 0.0776) and XBB recombinant (*p >* 0.05, *p =* 0.153).

### 3.1. Immune Escape of BA.5 and XBB Variants from BNT162b2 Double-Vaccinated Sera

Titres were determined against parental strain B.1, lineage B.1.1.529 BA.5 and recombinant XBB in order to quantify the antibody neutralisation immune escape of the mutated variants compared to the ancestral strain. Titres for the three lineages were compared using a one-way repeated-measures ANOVA test. Overall, both the BA.5 variant and XBB recombinant showed a significant immune evasion capacity compared to the ancestral B.1 strain, with noteworthy differences between the low- and medium-neutralising titre sera, on the one hand, and the high-neutralising sera, on the other.

**Figure 1:**
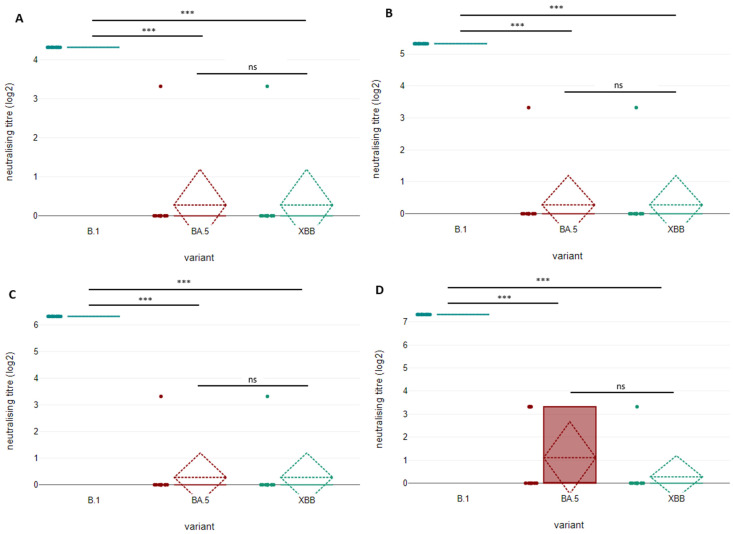
Comparison of neutralising antibody titres of low (titres 20 and 40) and medium (titres 80 and 160) neutralising sera against SARS-CoV-2 original strain B.1, lineage B.1.1.529 BA.5 and recombinant XBB at four weeks after the second dose of BNT162b2 vaccine. Sera were grouped depending on the neutralisation titre against ancestral lineage B.1 and retested with lineage BA.5 and recombinant XBB. For each group, 12 sera were tested (*n* = 12). Results for sera with a neutralisation titre of 20, 40, 80 and 160 are shown in panels (**A**), (**B**), (**C**) and (**D**), respectively. Neutralisation titre is expressed as the reciprocal of the highest serum dilution capable of inhibiting the appearance of a visible cytopathic effect. The detection limit of the assay is defined as a titre of 10; sera which did not reach the detection limit were considered negative and their titre was approximated to zero. The upper limit of detection is defined as a titre of 5120. Statistical comparison of neutralisation titres against the three considered viral variants were compared using a one-way repeated-measures ANOVA test (*** *p* < 0.01, ns = not significant). Medians are indicated with solid horizontal lines, means with dashed horizontal lines and standard deviations with dashed oblique lines.

Sera with a low (titres 20 to 40) to intermediate (titres 80 to 160) neutralising power against the ancestral strain showed a nearly complete escape from neutralisation, with only few sera in each group retaining a minimal neutralising capacity. Within the groups of sera with neutralising titres of 20 (*n* = 12), 40 (*n* = 12) and 80 (*n* = 12) against the original strain, only two samples for each class showed a residual neutralising activity against either the BA.5 variant or XBB recombinant. In any case, the neutralising titre was very low and never exceeded 10. Similarly, four sera with a neutralising titre of 160 (*n* = 12) against the ancestral strain retain a certain neutralisation against BA.5 variant, while only one of these sera is capable of neutralising XBB recombinant at the same dilution. In all these cases, the neutralising titre reported against either BA.5 or XBB was significantly lower than that originally shown for the ancestral strain, for each group of sera, with a significance at *p <* 0.01 (in every case, the *p* value resulted <0.00001). In all these sera groups, no statistically significant difference was reported between BA.5 and XBB at any of the two levels of statistical significance (*p* > 0.05) and both viral variants showed a similarly strong evasion from antibody neutralisation. The results of statistical comparisons among neutralising titres obtained when testing the low and intermediate neutralising sera with lineage B.1, lineage B.1.1.529 BA.5 and recombinant XBB are shown in Figure 1.

**Figure 2:**
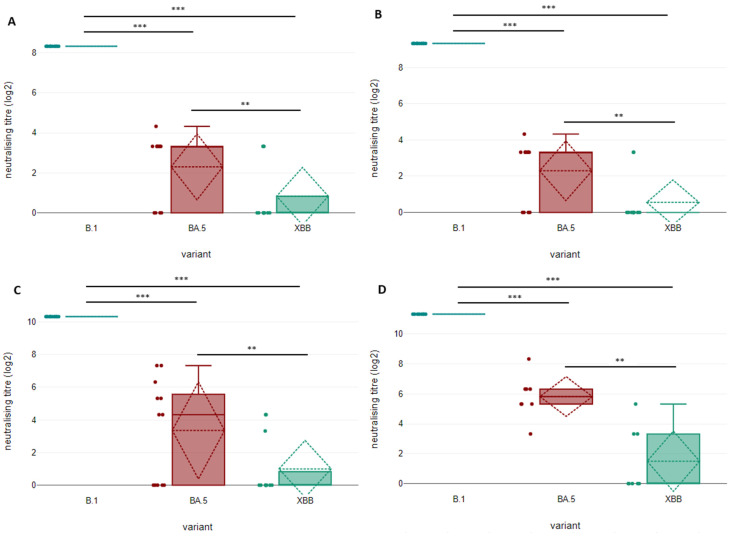
Comparison of neutralising antibody titres of highly neutralising sera (titres 320, 640, 1280 and 2560) against SARS-CoV-2 original strain B.1, lineage B.1.1.529 BA.5 and recombinant XBB at four weeks after the second dose of BNT162b2 vaccine. For the groups of sera with titres of 320, 640 and 1280, a total of 12 samples were tested (*n* = 12), while for the group of sera with a titre of 2560, only 8 samples were included (*n* = 8). Results for sera with a neutralisation titre of 320, 640, 1280 and 2560 are shown in panels (**A**), (**B**), (**C**) and (**D**), respectively. The detection limit of the assay is defined as a titre of 10; sera which did not reach the detection limit were considered negative and their titre was approximated to zero. The upper limit of detection is defined as a titre of 5120. Statistical comparison of neutralisation titres against the three considered viral variants were compared using a one-way repeated-measures ANOVA test (*** *p <* 0.01, ** *p <* 0.05). Medians are indicated with solid horizontal lines, means with dashed horizontal lines and standard deviations with dashed oblique lines.

For highly neutralising sera (titres 320, 640, 1280 and 2560) the difference between the ancestral strain and both mutated variants are statistically significant at *p <* 0.01. This is the same for low-to-medium neutralising titre sera. As previously stated, for the groups of sera with titres of 320, 640 and 1280, a total of 12 samples were tested (*n* = 12), while for the group of sera with a titre of 2560, only 8 samples were included (*n* = 8). In this case, the drop in neutralisation efficacy does not result in a complete escape of the mutated variants, but in a measurable and definable geometric mean titre (GMT) reduction. Moreover, unlike what was previously observed for low- and medium-neutralising sera, for high-neutralising sera, a statistical difference can be observed between BA.5 and XBB in all groups. More specifically, for sera with a neutralising titre of 320 against the ancestral strain, eight out of twelve sera show a neutralisation activity against BA.5, although this is strongly diminished if compared to the ancestral strain (seven sera only neutralise up to 1:10 dilution, while only one serum reaches 1:20), while only three sera neutralise the XBB recombinant at the minimum level (1:10). All in all, this results in a GMT reduction of 43-fold and 128-fold for BA.5 and XBB, respectively, compared to the ancestral strain, with XBB being approximately three times more resistant to antibody neutralisation if compared to BA.5. This translates into a statistically significant difference between BA.5 and XBB at *p <* 0.05 (*p =* 0.035). Similar neutralisation data were observed for sera with a neutralisation titre of 640 against the ancestral strain, with retained neutralisation never exceeding 1:20 dilution, resulting in a GTM reduction of 85-fold and 384-fold for BA.5 and XBB, respectively, compared to the ancestral strain. XBB, again, is 4.5 times more prone to neutralisation escape than BA.5 (difference between BA.5 and XBB significant at *p <* 0.05, *p =* 0.012). Conversely, sera with higher neutralisation capacity against the ancestral strain, i.e., 1280 and 2560, can maintain an overall substantial activity against BA.5 lineage (GMT reduction of 29-fold in both cases), which nonetheless results in being hampered against the XBB recombinant (GMT reduction of 307-fold and 341-fold for BA.5 and XBB, respectively). For these two classes of sera, the difference between BA.5 and XBB is wider, with XBB resistance to neutralisation increased by 10.4 to 11.5 times, respectively (significant at *p <* 0.05, *p =* 0.034 and *p =* 0.040, respectively). The results of statistical comparisons among neutralising titres obtained testing highly neutralising sera with lineage B.1, lineage B.1.1.529 BA.5 and recombinant XBB are shown in Figure 2.

### 3.2. Correlation between Anti-S1/S2 IgG Values and Neutralisation Titres against Ancestral and Mutated Strains

**Figure 3:**
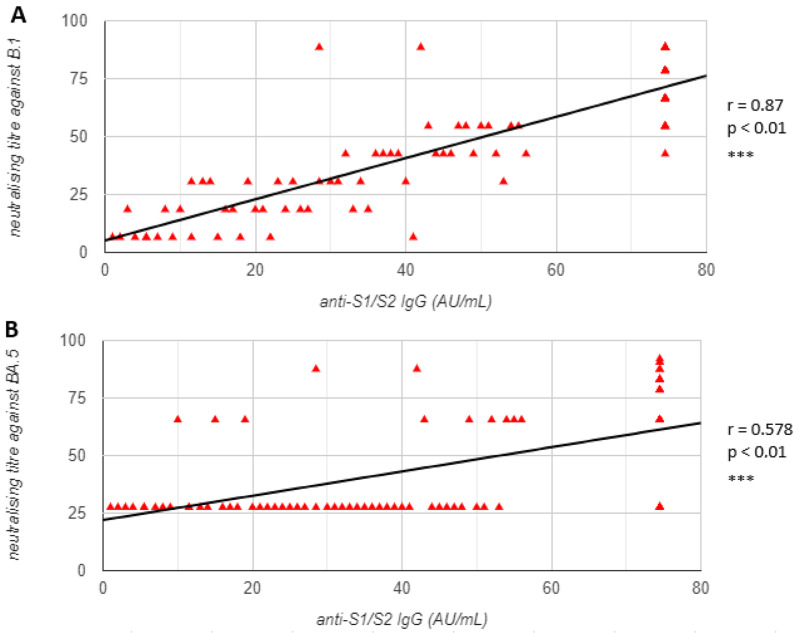

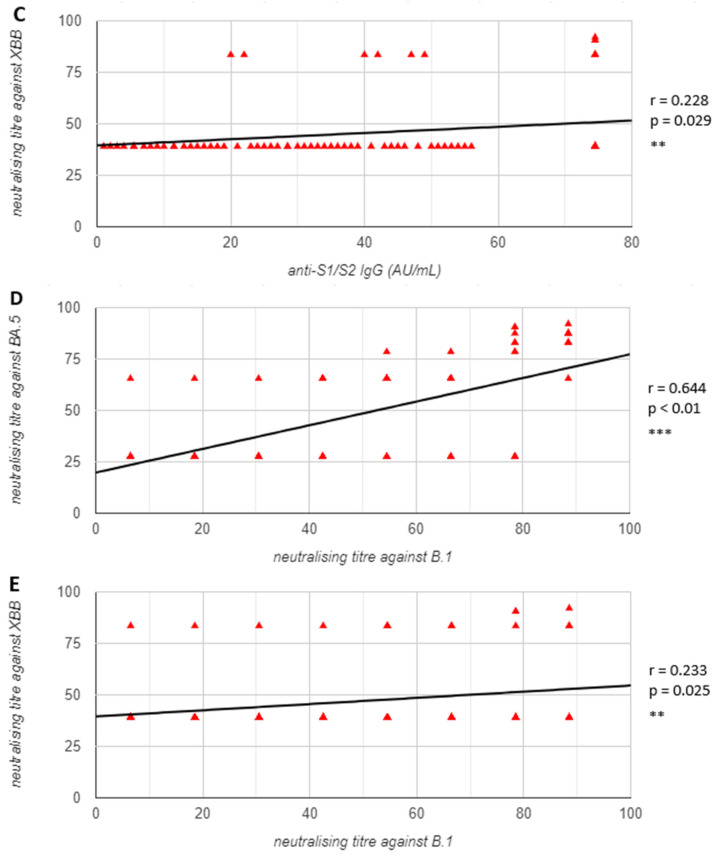
Correlation between anti-S1/S2 IgG values and neutralisation titres against ancestral strain B.1 and mutated lineage B.1.1.529 BA.5 and recombinant XBB, separately, as shown in panels (**A**), (**B**) and (**C**), respectively. Correlation between neutralising titre against lineage B.1 and both mutated variants was also analysed (panel (**D**) for B.1-BA.5 correlation and panel (**E**) for B.1-XBB correlation). Statistical correlation was tested using a non-parametric two-tailed Spearman’s rank correlation. Results r values and *p* values are reported in each graph (*** *p <* 0.01, ** *p* < 0.05).

Measured titres for each considered variant were later correlated with anti-S1/S2 IgG levels with a non-parametric two-tailed Spearman’s rank correlation. The results indicated that there is a significant large positive relationship between anti-S1/S2 IgG values and neutralising titres against lineage B.1 (r = 0.87, *p <* 0.01), as well as between IgG values and neutralising titre against BA.5 lineage (r = 0.578, *p <* 0.01). While the relationship between IgG values and neutralising titre against XBB recombinant the relationship is small, it is significant (r = 0.228, *p =* 0.029). The results suggest that the correlation between antibody levels and the protective function of a heavily mutated recombinant was partially disrupted by this antigenically distinct strain. This is also supported by the results of the Spearman correlation between the neutralising titre against B.1 and BA.5, which resulted in a large positive relationship (r = 0.644, *p <* 0.01) and between the neutralising titre against B.1 and XBB, which came back small but still significant (r = 0.233, *p =* 0.025). The results of the Spearman’s rank correlation are shown in Figure 3.

## 4. Discussion

Our data, together with a number of other studies analysing the immune status of adult individuals following the completion of a primary vaccine series, especially with mRNA vaccines, highlight a considerable heterogeneity concerning IgG and neutralising titres, in infection-naïve subjects if compared to infected, vaccinated subjects (not considered in our study) [21,22,23,24]. Specifically, the results for neutralising titres were low, especially against mutated variants. Correlation analysis between age, on the one hand, and IgG values and neutralising titres on the other, showed no statistical significance. On the contrary, a statistically significant difference in neutralising titres against BA.5 variant was noticed, while for the B.1 strain and XBB recombinant there was a statistically negligible heterogeneity. 

The emergence of novel circulating variants has raised significant concerns about the efficacy of containment interventions represented in part by vaccination campaigns. Plenty of existing studies offer the opportunity to investigate cross-neutralisation ability shown by vaccination-elicited antibodies against forthcoming viral variants. While some of these variants appear to be cross-neutralised with only slightly decreased potency compared to the ancestral wild-type strain, others have thus far demonstrated greater abilities to evade vaccine-elicited humoral immunity [25,26]. In the last few months, of greatest concern has been the emergence of novel viral strains resulting from the recombination of distinct parental lineages or sub-lineages within the same lineage. Among these, XBB, recombinant of BA.2.10.1 and BA.2.75 sub-lineages, which was first detected in the USA and Singapore in August 2022, has now climbed to a global prevalence of 5%, and has been detected in 52 countries across 6 continents [27,28]. This recombinant, with an approximate break point between spike mutations G446S and N460K, contains more receptor-binding domain mutations at antigenic sites than any other widespread circulating variant [28]. Despite the steady increase in prevalence, this variant has not yet been consistently associated with an increase in new infections, or an increased infection and correlated disease severity. Early evidence points nonetheless to an increased immune escape of this recombinant compared to other currently circulating Omicron sub-lineages, as a result of its specific set of mutations of the spike [13].

Taken together, our results show that both BA.5 and XBB are significantly more immune-evasive than the ancestral strain in subjects with a previous vaccination with BNT162b2, highlighting how this specific vaccine only achieves a partial cross-neutralization of novel variants. Regarding this, the XBB recombinant shows an even higher and statistically significant resistance to antibody-induced neutralisation than the BA.5 sub-lineage, at least in vitro, which may have contributed to its worldwide dissemination and to its unceasing, although slow, increase in prevalence. While our study only tested BA.5 and XBB immune evasion capacity from vaccine-elicited antibodies, it shows interesting and significant data, which point to the inability of these antibodies to effectively counteract viral infection, at least in vitro, especially in the presence of a low or moderate neutralising response, as determined by measuring their titres against the ancestral variants. On the contrary, a highly neutralising antibody response induced against the ancestral variant retained a certain, although diminished, neutralising activity against these mutated variants. These data suggest that these characteristics may increase the risk of vaccine breakthrough infection from XBB recombinant compared to previous sub-lineages.

One of the limitations of this study includes the relatively small sample size. This did not, however, invalidate the statistical soundness of the tests used to draw certain conclusions. This study is therefore to be considered exploratory, as it lays the foundations for further research on how neutralisation titres against these variants change over time and following other events such as booster doses or natural infection. In fact, currently, there are no data to document escape in different cohorts with a different history of vaccination and infection, such as in subjects with a past infection-induced antibody response (either caused by Omicron lineages or by distinct lineages) or in recipients of different types of vaccine. Moreover, we only assessed immune evasion capability of these novel variants against B-cell responses, which seems to be less affected by mutation determining humoral response escape [29]. Determining the extent to which these specific variants, along with forthcoming ones, escape cellular immunity constitutes a further possible development in the field. 

Altogether, we were able to demonstrate an impairment in the neutralising antibody response induced by two doses of the BNT162b2 mRNA-vaccine, which differentially blocked diverse variant strains. The study highlights an enhanced immune escape ability of the XBB recombinant, generally leading to almost abolished antibody protection against mutated variants, which could be of great interest, not only in the general population but especially among healthcare workers, due to direct professional exposure. These data support the importance of immune escape monitoring of new and forthcoming variants to better tailor future vaccination strategies. Reformulation of existing vaccines to include diverse spike sequences may also be needed, so that they are able to elicit a broader antibody response to heavily mutated variants, in order to better respond to future viral evolution.

## Data Availability

All data supporting the findings of this study are discussed in the main text and summarized in graphs.
